# Endobronchial ultrasound guided fine needle aspiration versus transcervical mediastinoscopy in nodal staging of non small cell lung cancer: a prospective comparison study

**DOI:** 10.1186/1749-8090-7-51

**Published:** 2012-06-06

**Authors:** Ruoyu Zhang, Christina Mietchen, Marcus Krüger, Bettina Wiegmann, Heiko Golpon, Sabine Dettmer, Axel Haverich, Patrick Zardo

**Affiliations:** 1 Department of Cardiac, Thoracic, Transplantation and Vascular Surgery, Hannover Medical School, Carl-Neuberg-Str. 1, Hannover, 30625, Germany; 2 Department of Pneumology, Hannover Medical School, Carl-Neuberg-Str. 1, Hannover, 30625, Germany; 3 Department of Radiology, Hannover Medical School, Carl-Neuberg-Str. 1, Hannover, 30625, Germany; 4 Department of Cardiac, Thoracic, Transplantation and Vascular Surgery, Hannover Medical School, Carl-Neuberg Str. 1, Hannover, 30625, Germany

**Keywords:** EBUS-FNA, Mediastinoscopy, NSCLC, Nodal staging

## Abstract

**Background:**

At present only few studies directly compare the diagnostic yield of endobronchial ultrasound guided fine needle aspiration (EBUS-FNA) and transcervical video-assisted mediastinoscopy (TM) for mediastinal lymph node staging in patients with NSCLC. If and when EBUS-FNA may replace TM as Gold Standard remains controversial.

**Methods:**

From April 2008 to December 2009, 36 patients with mediastinal lymphadenopathy underwent simultaneous EBUS-FNA/ TM at our institution. Among them were 26 patients with confirmed or suspected NSCLC.

**Results:**

A total of 133 samples were obtained by EBUS-FNA and 157 samples by TM. EBUS-FNA achieved significantly less conclusive, but more indeterminate pathological results in comparison to TM (78.7% vs. 98.6%, *p* < 0.001; 14.9% vs. 1.4%, *p* = 0.007). Less paratracheal nodes were sampled by EBUS-FNA (right: 46.2% vs. 88.5%, *p* = 0.003; left: 23.1% vs. 65.4%, *p* = 0.005), while sampling rates in the subcarinal localisation were comparable (96.2% vs. 80.8%, *p* = NS). Among patients with confirmed NSCLC and conclusive EBUS-FNA/ TM findings (n = 18), the prevalence of N2/N3 disease was 66.7% (n = 12) according to TM findings. Diverging nodal stages were found in five patients (27.8%). Three patients who were N2 negative in EBUS-FNA were upstaged to N2 or N3 by TM, two patients with N2 status in EBUS-FNA were upstaged to N3 by TM.

**Conclusions:**

Compared to TM, EBUS-FNA had a lower diagnostic yield and resulted in systematic mediastinal nodal understaging. At this point we suggest corroborating negative EBUS-FNA results by transcervical mediastinoscopy.

## Background

Accurate mediastinal nodal assessment is crucial to stratify patients with NSCLC for adequate therapy, including (neo)adjuvant and definitive treatment protocols or primary curative resection. Though transcervical mediastinoscopy (TM) remains the gold standard for mediastinal nodal staging, endobronchial ultrasound guided fine needle aspiration (EBUS-FNA) has gained widespread acceptance as alternative diagnostic modality in recent years [1-4]. Literature reviews and meta-analyses of EBUS-FNA in lung cancer staging show a sensitivity of 88% to 93%, as well as a specificity of 100% [5,6]. Current American College of Chest Physicians (ACCP) guidelines for invasive mediastinal staging of lung cancer acknowledge that EBUS-FNA has a similar sensitivity although a higher false negative rate when compared to video-assisted mediastinoscopy (90% vs. 90% and 24% vs. 10%, respectively) [7].

Hitherto, it is still controversial if and when EBUS-FNA may replace TM as gold standard in mediastinal nodal assessment prior to curative lung resection. Very few published studies directly compare the diagnostic value of EBUS-FNA and TM in this regard. While certain groups report lower sensitivity and similar specificity of EBUS-FNA [8], other postulate higher sensitivity and negative predictive value of EBUS-FNA [9].

We launched a prospective trial set to compare the diagnostic value of EBUS-FNA and TM for mediastinal nodal staging in patients with confirmed or suspected NSCLC by performing both procedures concomitantly.

## Methods

**Figure 1:**
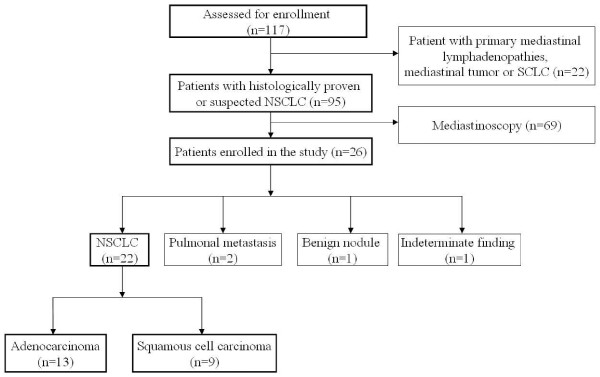
Chartflow demonstrating patient enrollment and final diagnosis.

From April 2008 to December 2009, 117 patients were screened for enrolement in the present study. Our inclusion criteria consisted of histologically proven or suspected NSCLC, eligibility for lung resection and adult age (> 18 years). The exclusion criteria included distant metastasis, neoadjuvant therapy, N2-bulky disease, previous mediastnoscopy, pregnancy, coagulation or platelet function disorder, ongoing anticoagulation therapy. A total of 26 consecutive patients were subsequentially enlisted and underwent concomitant EBUS-FNA/ TM for mediastinal nodal assessement in accordance with current ESTS guidelines after multidisciplinary tumour board approval. Sixty-nine patients were excluded due to refusal of simultaneous EBUS-FNA and TM. Enrolement modalities and final diagnoses are presented as chartflow (Figure 1). Standard diagnostic workup prior to simultaneous EBUS-FNA/TM included medical history, phyiscal examination, laboratory testing, bronchoscopy, cMRI and PET/CT. This study conforms to the principles outlined in the Declaration of Helsinki and approval of our IRB was obtained. Every patient was informed about all procedures and gave his/her written informed consent.

All patients underwent simultaneous EBUS-FNA and TM by a single thoracic surgeon experienced in both procedures. After induction of general anaesthesia and orotracheal intubation, EBUS-FNA was performed using a flexible ultrasound bronchoscope (Olympus BF − UC180F, Olympus Medical Systems Europe, Hamburg, Germany) connected to an ultrasound processor (Olympus EU − C60). Cytological specimens were obtained by means of 22-guage needles for transbronchial aspiration (Olympus NA−201 SX-4022, Olympus Medical Systems Europe, Hamburg, Germany). The technique was similar to that previously described by Yasufuku et al. [10]. Mediastinal and hilar lymph nodes (LN) were systematically examined and sampled with at least 3 passes at each visualized LN under direct ultrasound guidance. To prevent contamination, different needles were used for each LN station. LN was sampled in order of N3, N2 and then N1. Afterwards, transcervical mediastinoscopy was performed using a standard video-mediastinoscope (Storz, Germany) according to the standard technique first described by Carlens. Both our cytologist and pathologist were blinded, and rapid cytological evaluation or intraoperative frozen section analysis were available only in case of highly suspect N3 nodes. Otherwise no information on cytological and histological findings was passed on intraoperatively.

Descriptive statistics are presented as mean ± standard deviation. Categorical variables are expressed as percentages and evaluated with Fischer’s exact test. Continuous data were compared using the student *t*-test. Statistical significance was assumed if p < 0.05. All statistical evaluation was performed using SPSS (version 16.0 for Windows; SPSS, Inc., Chicago, IL).

## Results

A total of 26 patients (64.5 ± 11.3 years, 12 males) were enrolled in this study. All showed cT1 and cT2 tumour lesions except for one patient with a cT3 tumour. Results of preoperative clinical mediastinal nodal staging are listed in Table 1.

**Table 1:** Results of preoperative clinical mediastinal nodal stages (n = 26)

**Clinical N staging**	
cN0	3 (11.5%)
cN1	6 (23.1%)
cN2	14 (53.8%)
cN3	3 (11.5%)

Analyses of all sampled LN stations are presented in Table 2. A total of 133 samples from 47 LN stations were obtained by EBUS-FNA as well as 157 samples from 71 LN stations by TM. The size of LN sampled by EBUS-FNA was 11.1 ± 6.1 mm. Compared to TM, there were fewer LN stations sampled by EBUS-FNA, but more samples per LN station were obtained. In terms of pathological findings, EBUS-FNA achieved significantly less conclusive, but more indeterminate results in comparison to TM. Non-representative samples were only found in EBUS-FNA (n = 3).

**Table 2:** Analysis of all sampled lymph node stations

	**EBUS-FNA**	**Mediastinoscopy**	***p*****value**
Sampled LN stations	47	71	
Total sample number	133	157	
Sampled LN stations per patient	1.7 ± 0.9	2.7 ± 1.0	0.001
Samples per LN station	2.8 ± 1.6	2.2 ± 1.3	<0.001
Conclusive finding	37 (78.7%)	70 (98.6%)	<0.001
Indeterminate finding	7 (14.9%)	1 (1.4%)	0.007
Non-representative material	3 (6.4%)	0 (0%)	0.061

A detailed analysis of sampled mediastinal LN is presented in Table 3. EBUS-FNA achieved biopsy of paratracheal LN in fewer patients compared to TM. Fewer samples were also obtained by EBUS-FNA at these LN stations. The subcarinal station was sampled in more patients by EBUS-FNA, however without statistical significance. More samples were obtained by EBUS-FNA at the subcarinal level. Among patients with confirmed NSCLC who had both conclusive EBUS-FNA and TM findings (n = 18), prevalence of N2/N3 disease was 66.7% (n = 12) according to TM findings. Diverging nodal stages were found in five patients (27.8%). Three patients who were N2 negative in EBUS-FNA were upstaged to N2 or N3 by TM, two patients with N2 status in EBUS-FNA were upstaged to N3 by TM (Table 4). Understaging resulted from false negative findings in EBUS-FNA in three patients. In two patients paratracheal LN stations were not sampled by EBUS-FNA, also leading to nodal understaging.

**Table 3:** Analysis of mediastinal lymph nodes

	**EBUS-FNA**	**Mediastinoscopy**	**P value**
Total samples of N2 nodes	128	157	
Sample of N2 nodes per patient	2.7 ± 1.6	2.2 ± 1.3	0.040
Sampling rate			
Right paratracheal	46.2% (n = 12)	88.5% (n = 23)	0.003
Left paratracheal	23.1% (n = 6)	65.4% (n = 17)	0.005
Subcarinal	96.2% (n = 25)	80.8% (n = 21)	0.191
Samples per station			
Right paratracheal	1.3 ± 0.3	2.1 ± 0.4	0.002
Left paratracheal	1.4 ± 0.3	1.6 ± 0.3	0.058
Subcarinal	1.8 ± 0.4	1.2 ± 0.2	0.007
Pathological N-staging*			
Negative N2/3	9	6	
N2	7	7	
N3	2	5	

* n = 18 patients with confirmed NSCLC and both conclusive EBUS-FNA and TM findings.

**Table 4:** Discordant mediastinal nodal staging between EBUS-FNA and TM

**Pts**	**EBUS-FNA**		**Mediastinoscopy**	
	**Stage**	Results of single LN stations*	**Stage**	Results of single LN stations*
#2	N2 neg.	neg. (2), neg. (4), neg. (3)	N3	neg. (2), pos. (1), pos. (2)
#11	N2 neg.	neg. (2), neg. (2), neg. (2)	N2	pos. (7), NS, NS
#15	N2	pos. (2), pos. (3), neg. (1)	N3	NS, pos. (4), pos. (3)
#19	N2	NS, pos. (2), NS	N3	pos. (6), pos. (2), pos. (1)
#20	N2 neg.	NS, neg. (7), NS	N2	pos. (2), neg. (2), NS

* The results of single LN stations are presented in order of right paratracheal, subcarinal and left paratracheal station. The sample number of single LN station is presented in the parasenthesis. NS = not sampled.

Neither EBUS-FNA nor TM lead to postoperative complications. Over our observation period, nine patients underwent curative lung resection with systematic radical lymphadenectomy. The postsurgical LN staging was in accordance to TM results in all patients. Three patients received adjuvant chemotherapy due to single station N2 disease determinated by both TM and postsurgical pathological examination. A high prevalence of N2/N3 disease allowed for postsurgical LN staging only in nine out of 26 patients. This prevented us from extrapolating sensitivity, specificity, diagnostic accuracy and false-negative rate for each procedure.

## Discussion

In the present study, EBUS-FNA and TM were directly compared for mediastinal nodal assessment in patients with proven or suspected NSCLC. In this series, EBUS-FNA had a lower diagnostic yield and resulted in systematic understaging of mediastinal LN metastases, mainly due to false negative findings. These results are generally in line with recent meta-analyses evaluating the diagnostic value of EBUS-FNA for preoperative mediastinal LN staging in lung cancer patients. Among them is a meta-analysis on which the current ACCP guidelines are based, where EBUS-FNA had a false negative rate as high as 24% [7]. A further meta-analysis of Gu and co-workers acknowledged that EBUS-FNA achieves an overall sensitivity of 76% in all patients, including those with negative pre-operative CT and/or PET/CT scans [6]. In a current study of Defranchi and coworkers, 29 patients with suspected or confirmed lung cancer had negative EBUS-FNA findings and underwent subsequent mediastinoscopy. TM discerned metastatic nodes in eight of these patients (28%) [11].

In recent years EBUS-FNA has gained widespread acceptance as an alternative diagnostic modality for mediastinal staging in NSCLC. Despite technical improvements (real-time ultrasound guidance) and adequate techniques, the inherent limit of small tissue volumes obtainable through FNA-procedures persists. Small metastatic tumour deposits are more likely to be missed by needle aspiration than by extensive biopsy. As pointed out by Dr. Shrager in his comment, a needle-based technique can hardly achieve the same reliability than a surgical procedure that allows for extensive tissue sampling [12]. These findings are corroborated by various studies of conventional transbronchial needle aspiration in mediastinal LN staging demonstrating that 19-gauge needles, which provide a core of tissue and allow for histological evaluation, are more sensitive than thinner cytology needles (21- or 22-gauge) [13,14].

Recent reports on comparable or even higher sensitivity of EBUS-FNA are mostly based on analyses of enlarged mediastinal LN in cohorts with high prevalence of malignancy. In a study of Ernst et al. comparing EBUS/FNA and TM in n = 66 patients, only radiologically enlarged mediastinal LN (mean short axis 15 mm) were sampled and analyzed [9]. Certain factors may have biased their study in favour of EBUS-FNA: While TM was performed by eight different surgeons EBUS-FNA was performed by just three endoscopists. Secondly, an unusually high rate of non-diagnostic procedures (25.8%, 17 out of 66 patients) was encountered in their study. Additionally, they found a significantly higher accuracy of EBUS-FNA in subcarinal LN stations compared to TM, while diagnostic rates from paratracheal LN did not differ between both procedures and explained this observation by postulating that posterior subcarinal LN are beyond reach of mediastinoscopy. This diverges from our findings, as we had comparable sampling rates in subcarinal LN for EBUS-FNA and TM. Finally, some EBUS-FNA cases were performed within one week prior to TM, potentially rendering subsequent LN sampling more difficult.

Mediastinal nodal staging in NSCLC is initially based on the results of conventional imaging. To confirm nodal involvement in radiologically enlarged mediastinal LN, either EBUS-FNA or TM are reasonable [4,7]. Even in this patient subset, negative results from EBUS-FNA should be corroborated by TM. At present EBUS-FNA does not appear to be reliable enough to categorically rule out metastatic involvement of normal-sized mediastinal LN. Additionally, paratracheal LN, particularly on the left side, are technically difficult to visualize by EBUS-FNA and small LN size and adherence to vessel wall render sampling generally challenging [15]. In 2007, Yasufuku et al. reported preliminary results of a prospective controlled trial comparing EBUS-FNA and mediastinoscopy for mediastinal nodal staging of lung cancer. EBUS-FNA followed by TM was performed in 33 patients with confirmed or suspected NSCLC to determine suitability for surgical resection. The mean short axis of the biopsied LN was 6.7 mm. EBUS-FNA achieved lower sensitivity, but comparable specificity and diagnostic accuracy when compared to TM (76.9% vs. 84.6%, 100% vs. 100% and 90.9% vs. 93.9%, respectively). A discordant mediastinal LN staging was found in five patients (15.3%) [8]. During the 2011 AATS Meeting, the same group presented updated results from their ongoing study based on 153 patients. They found no significant differences between EBUS-FNA and TM in the diagnostic yield for mediastinal nodal staging in NSCLC. The sensitivity, specificity, negative predictive value and diagnostic accuracy for mediastinal lymph node staging for EBUS-TBNA and MS were 84.3%, 100%, 92.7%, 94.8% and 86.3%, 100%, 93.6%, 95.4%, respectively [16]. This very interesting development suggests that EBUS-FNA may achieve the same diagnostic value as TM in mediastinal nodal staging in very experienced hands. It should be kept present that those results were reported by leading experts in the field.

The authors do recognize various limitations of the present study. They primarily include an inherent single investigator bias and a modest sample size. On the other hand, an interpersonal bias, which was common to various previous comparative studies, was avoided.

## Conclusions

Our current results suggest that EBUS-FNA still has a lower diagnostic yield than TM and may lead to systematic nodal understaging in patients with suspected or confirmed NSCLC. At present EBUS-FNA appears as not reliable enough to safely rule out mediastinal nodal metastases and we therefore encourage to still corroborate a negative EBUS-FNA by transcervical mediastinoscopy.

## Abbreviations

EBUS-FNA, Endobronchial ultrasound guided fine needle aspiration; NSCLC, Non small cell lung cancer; TM, Transcervical video-assisted mediastinoscopy; ACCP, American College of Chest Physicians; ESTS, European Society of Thoracic Surgeons; cMRI, Cerebral magnetic resonance imaging; PET/CT, Prositron-emission tomography/computer tomography.

## Competing interests

All authors have no financial or other interests regarding the submitted manuscript.

## Authors’ contributions

RZ and CM carried out the conception and design of the study, acquisition of data, analysis and interpretation of the data, statistical analysis as well as drafting of the manuscript. MK participated in the analysis and interpretation of the data, drafting of the manuscript and critical revision of the manuscript. BW and SD participated in the acquisition of data. HG and AH participated in the analysis and interpretation of the data, critical revision of the manuscript and supervision of the study. PZ performed surgeries, participated in the conception and design of the study, acquisition and interpretation of the data, drafting and critical revision of the manuscript. All authors read and approved the final manuscript.
